# Adult Attachment Style, Emotion Regulation, and Social Networking Sites Addiction

**DOI:** 10.3389/fpsyg.2019.02352

**Published:** 2019-10-24

**Authors:** Chang Liu, Jian-Ling Ma

**Affiliations:** ^1^Yangtze Normal University, Chongqing, China; ^2^Chongqing University of Posts and Telecommunications, Chongqing, China

**Keywords:** adult attachment style, attachment anxiety, attachment avoidance, emotion regulation, SNS addiction

## Abstract

While there is substantial evidence that emotion regulation plays a role in the maintenance of substance and behavior addiction, its role in addiction to social networking sites (SNS) remains unclear. Drawing on attachment theory, we explore whether emotion regulation mediates the relationship between insecure attachment and SNS addiction among 463 college students. The participants completed the short version of the Experience in Close Relationships Scale, Difficulties in Emotion Regulation Scale, and Chinese Social Media Addiction Scale. The results indicated that attachment anxiety positively predicted SNS addiction and that emotion regulation mediated this link. These findings suggest that individuals’ affective regulation capability should be a target of future interventions and treatments.

## Introduction

The use of social networking sites (SNS) is now pervasive among college students because of the widespread availability of mobile devices. According to the latest official report released by the China Internet Network Information Center (CNNIC, 2019), there are almost 0.829 billion Netizens in China. SNSs are centered on the formation and maintenance of social connections and close relationships. The top three most popular social media sites in China were WeChat (utilization ratio: 85.5%), QQ (utilization ratio: 67.8%), and Sina Weibo (utilization ratio: 37.1%). WeChat is a Chinese multipurpose messaging, social media, and mobile payment app. It was first released in 2011; by 2018, it was one of the world’s largest standalone mobile apps by monthly active users (over 1 billion). Like WeChat, QQ is another well-known social media platform consisting of online social games, music, shopping, microblogging, movies, and group and voice chat software, with a longer history than WeChat. Sina Weibo implements many features from Twitter (i.e., people follow celebrities).

Although SNSs have clear benefits, e.g., for broadening relationships, relaxation, and overcoming physical limitations, the excessive use of SNS—also known as SNS addiction—can have a detrimental effect on health. Excessive use of SNS leads to higher level of envy, anxiety, and depression (Tandoc et al., 2015; Appel et al., 2016; Liu and Ma, 2018a, b; Keles et al., 2019). SNS addiction can be defined as a failure to control compulsive use of SNSs despite the obvious negative consequences (Kuss and Griffiths, 2017). Numerous studies have explored the personality factors associated with SNS addiction, with attachment style being a particularly significant predictor (Flynn et al., 2018; Worsley et al., 2018a, b; Chen, 2019). However, the mechanism underlying this association remains unclear. In the present study, we investigated emotional regulation difficulties as a possible mediator of this association.

Adult attachment style is formed through early interactions between an infant and one or more primary caregivers (Bowlby, 1973, 1980, 1982, 1988). Attachment theory holds that early emotional bonds between infants and caregivers aid in the formation of infants’ internal working model of self and social interactions (Bretherton, 1987). This model is relatively stable and carries into adulthood, and thereby forms the basis of close and romantic relationships (Hazan and Shaver, 1987; Feeney and Noller, 1990; Bartholomew and Horowitz, 1991). When a caregiver is perceived as responsive, accessible, and trustworthy, an infant develops a secure attachment style. However, when the primary caregiver is inconsistent, unavailable and/or unresponsive, it can give rise to a negative internal working model and insecure attachment (Bretherton, 1987). Currently, scholars acknowledge two major dimensions of adult insecure attachment: attachment anxiety and avoidance (Brennan et al., 1998). An anxious attachment style (i.e., having high attachment anxiety) is characterized by hyperactivity of the attachment system, leading to a constant need to seek support and comfort. An avoidant attachment style, on the other hand, is characterized by a deactivated attachment system, which leads to continual inhibition of psychological and social relationship needs, self-reliance, and a marked distance from others. As far as the association between attachment and SNS addiction concerned, prior studies found that attachment anxiety positively predicts, while avoidance negatively predicts, the addiction (Blackwell et al., 2017; Worsley et al., 2018a). This is reasonable, given that the primary function of SNS to foster and maintain social ties, and to obtain comfort and social support (D’Arienzo et al., 2019). Moreover, other investigations reveal that well-being (Worsley et al., 2018b) and needs satisfaction (Chen, 2019) mediated the attachment-SNS addiction association.

Attachment theory has been conceptualized as an emotion regulation theory (Mikulincer et al., 2003; Schore and Schore, 2008). When an infant does not experience reliable or consistent protection and support from its primary caregiver(s), it develops a hyperactivation or deactivation model of the attachment system to obtain attachment-directed goals, which is partially done via the process of emotion regulation (Cassidy, 1994). Emotion regulation refers to the act of altering one’s emotional experiences via the initiation, maintenance, or modification of their frequency, intensity, or duration (Kobak et al., 1993). A hyperactivation attachment model (i.e., attachment anxiety) develops when a caregiver cannot engage in consistent, sensitive, or responsive interactions to their infant. Individuals with higher levels of anxious attachment tendency are inclined to up-regulate their emotions, thereby overreacting and maintaining high levels of negative emotion (Mikulincer et al., 2003). Deactivating strategies are characteristic of people scoring relatively high on the attachment avoidance dimension. Attachment avoidance is associated with low levels of intimacy and emotional involvement in close relationships, suppression of painful thoughts, repression of negative memories, lack of cognitive accessibility to negative self-representations, projection of negative self-traits onto others, failure to acknowledge negative emotions, and denial of basic fears (Kobak et al., 1993).

In fact, numerous studies have found that attachment anxiety and emotional dysregulation are positively correlated, and these two variables also appear to contribute to maladaptive behavior and affective problems, such as problematic internet use (Estevez et al., 2018), eating disorders (Norrish et al., 2019), anxiety (Esbjorn et al., 2012; Nielsen et al., 2017), and depression (Marques et al., 2018; Owens et al., 2018; Read et al., 2018). Difficulties in emotion regulation are in particular considered risk factors for both substance (e.g., alcohol and drug abuse) and behavioral addiction (e.g., gambling disorder, video game addiction, and smartphone addiction). For example, the internet might serve as a way for individuals to escape feelings of distress and cope with stress, anxiety, and depression. The association between emotion regulation and SNS addiction has also been confirmed, that is, greater difficulties in emotion regulation are associated with problematic use of SNS, as assessed using modified measures of substance abuse (Hormes et al., 2015). Dysfunctional emotional regulation has also been found to be correlated with (Pontes et al., 2018) and predict problematic Facebook use (Marino et al., 2019).

Given the empirical and theoretical evidence of the associations between (a) insecure attachment style and SNS addiction, (b) insecure attachment style and emotional dysregulation, and (c) emotional dysregulation and SNS addiction, it is reasonable to assume that insecure attachment promotes maintenance of emotional dysregulation, which in turn gives rise to SNS addiction. To our knowledge, this model has not yet been tested.

**FIGURE 1:**
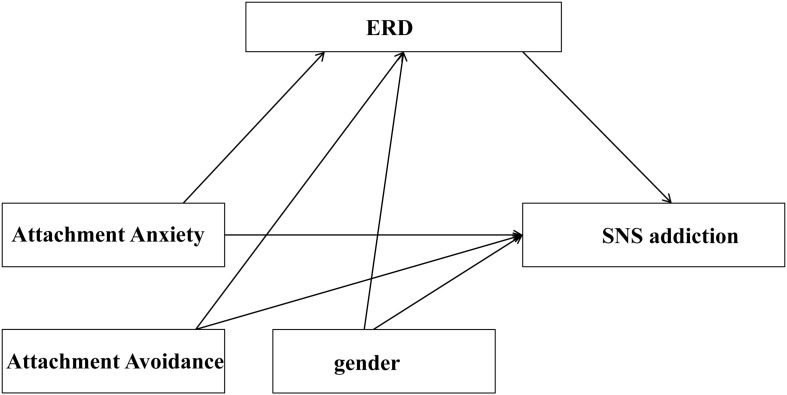
The hypothesized conceptual model of present study. When attachment anxiety is a predictor, attachment avoidance and gender were covariates. When attachment avoidance is a predictor, attachment anxiety and gender were covariates.

Therefore, the aim of the present study was to determine whether there is an indirect relationship between insecure attachment and SNS addiction via emotion regulation. We used cross-sectional data gathered from a sample of Chinese college students. The following hypotheses were proposed: (1) attachment anxiety positively predicts SNS addiction, while attachment avoidance negatively predicts it; and (2) the association between insecure attachment and SNS addiction is mediated by emotional regulation dysfunction. Figure 1 shows the hypothesized model of our study.

## Materials and Methods

### Participants

A total of 463 college students in China (344 female, M ± SD = 19.94 ± 1.11, range = 17–24 years) volunteered and completed a questionnaire survey of paper-pencil version. All the participants have SNS use experiences at least half a year.

### Measures

#### Experiences in Close Relationship Scale Short Version

The Experiences in Close Relationship Scale short version (ECR-SV; Wei et al., 2007) is a twelve-item measure for evaluating adult attachment. The scale is comprised of two six-item subscales: anxiety and avoidance. Each item is rated on a 7-point Likert scale ranging from 1 = strongly disagree to 7 = strongly agree. The ECR-SV has been found to have good psychometric properties (both reliability and validity) in an Asian sample (Lee and Shin, 2019). The Cronbach’s α values in our study were 0.84 for the anxiety scale and 0.747 for the avoidance scale.

#### 16-Item Version of the Difficulties in Emotion Regulation Scale

The 16-item version of the Difficulties in Emotion Regulation Scale (DERS-16) (Bjureberg et al., 2016) covers five dimensions: (1) non-acceptance, or the lack of acceptance of emotional responses; (2) goals, or difficulty engaging in goal-oriented behaviors; (3) impulse, or difficulty in controlling impulses; (4) strategies, or lack of access to emotion regulation strategies; and (5) clarity, or lack of emotional clarity. The items are rated on a scale of 1 = *almost never* to 5 = *almost always*. In a previous study, the DERS-16 was found to have high internal consistency (α = 0.92) and a significant and positive correlation with another measure of emotion regulation (Bjureberg et al., 2016), demonstrating its validity. In our sample, the total scale had excellent internal consistency (Cronbach’s α = 0.93), while the five subscales demonstrated acceptable values: non-acceptance, Cronbach’s α = 0.760; goals, α = 0.777; impulse, α = 0.849; strategies, α = 0.846; and clarity, α = 0.725.

#### Chinese Social Media Addiction Scale

The Chinese Social Media Addiction Scale (Liu and Ma, 2018a, b) consists of 28 items divided into six dimensions: preference for online social interaction, mood alteration, negative consequences of continued use, compulsive use and withdrawal, salience, and relapse. Each item is rated on a 5-point Likert-type scale (1 = strongly disagree, 5 = strongly agree), with higher scores indicating a higher level of SNS addiction. In the present study, the Cronbach’s α value was 0.944.

### Procedure

All the data were collected in August 2018. Approval for the study was obtained from the Human Research Ethics Committee of Chongqing University of Posts and Telecommunications. Written informed consent was obtained from all participants above the age of 16 and from the parents of participants below the age of 16. They were informed that their participation was voluntary and that they could terminate participation anytime they wanted. Participants received no rewards for their participation.

### Statistical Analysis

Descriptive statistics and correlation analysis were conducted with SPSS Statistics 20.0 (IBM Corp., Armonk, NY, United States). The proposed mediation model was then tested using the PROCESS macro model 4 (Hayes, 2013). We used the bootstrapping method (with 5000 resamples) to produce 95% bias-corrected confidence intervals (CI) of the model effects. CI that did not include zero indicated significant effects (α = 0.05).

## Results

The descriptive results are shown in Table 1 and Supplementary Table S1. Both attachment anxiety and avoidance were positively correlated with emotion dysregulation, which in turn was correlated with SNS addiction. Moreover, anxiety and avoidance were correlated with each other significantly. However, the relationship between attachment avoidance and SNS addiction was not significant.

**TABLE 1:** Descriptive statistics and results of correlational analysis of variables (*N* = 463).

	**M**	**SD**	**1**	**2**	**3**	**4**
1. Attachment anxiety	21.85	4.93	1			
2. Attachment avoidance	21.87	4.73	0.171^∗∗^	1		
3. Emotion regulation	39.28	12.03	0.413^∗∗^	0.122^∗∗^	1	
4. Social networking sites addiction	77.81	19.39	0.388^∗∗^	0.065	0.478^∗∗^	1

*^∗∗^*p* < 0.01.*

Model 4 of the PROCESS macro was used to test the mediating role of emotional dysregulation in the associations of insecure attachment (anxiety and avoidance) and SNS addiction. Since gender is not balanced in this study, it may contribute to the measures. Thus, *t*-tests were performed on the four measures to test if there is any contribution from gender. The results showed that there are no gender difference concerning attachment anxiety (*M*_male_ = 22.58, *M*_female_ = 21.60, *t* = 1.86, *p* = 0.06) and avoidance (*M*_male_ = 21.84, *M*_female_ = 21.87, *t* = −0.06, *p* = 0.95), but there are significant gender differences for emotional regulation difficulties: (*M*_male_ = 41.99, *M*_female_ = 38.34, *t* = 2.87, *p* < 0.001) and SNS addiction (*M*_male_ = 83.03, *M*_female_ = 76, *t* = 3.45, *p* < 0.01). To obtain the independent effects of attachment anxiety and avoidance on SNS addiction, we included gender as well as avoidance/anxiety as covariate for overall anxiety and avoidance.

Table 2 shows the effect of attachment anxiety on SNS addiction through emotional regulation difficulty. The total effect of attachment anxiety on SNS addition was 1.48 (95% CI = 1.14–1.86). Attachment anxiety significantly predicted emotion dysregulation (*b* = 0.963, *p* < 0.001), which in turn significantly predicted SNS addiction (*b* = 0.6, *p* < 0.001). The residual direct effect was also significant (*b* = 0.9, *p* < 0.001). The indirect effect = 0.58, 95% CI = 0.37–0.82, the effect size = 0.58/1.48 = 0.39, 95%CI = 0.24–0.59. Emotion dysregulation therefore played a partial mediating role in the association between attachment anxiety and SNS addiction. This model accounted for 28.1% of the variance in SNS addiction.

**TABLE 2:** The Influence of attachment anxiety on SNS Addiction with gender and avoidance as covariates.

	**Consequent**					
**Antecedent**	**Model 1 (ERD)**			**Model 2 (SNS addiction)**		
	**Coefficient**	**SE**	***p***	**Coefficient**	**SE**	***p***
Anxiety attachment	0.963	0.11	<0.001	0.9	0.17	<0.001
ERD	–	–	–	0.6	0.07	<0.001
Gender	2.71	1.17	<0.05	3.94	1.77	<0.05
Avoidance attachment	0.14	0.11	=0.2	−0.08	1.65	=0.62
Constant	15.32	2.46	<0.001			
	*R*^2^ = 0.18			*R*^2^ = 0.281		
	*F*(3,459) = 34.2, *p* < 0.001			*F*(4,458) = 44.7, *p* < 0.001		

*ERD: emotional regulation difficulty.*

Table 3 shows the effect of attachment avoidance on SNS addiction through emotion regulation difficulty. The total effect of attachment avoidance on SNS addition was 0.0045 (95%CI = −0.344–0.35). Avoidance attachment predicted emotion dysregulation nonsignificantly (*b* = 0.14, *p* = 0.2), which in turn predicted SNS addiction significantly (*b* = 0.6, *p* < 0.001). The residual direct effect was not significant (*b* = −0.08, *p* = 0.63). Emotion dysregulation therefore did not mediate the association between attachment avoidance and SNS addiction (indirect effect = 0.09, 95%CI = −0.05–0.22).

**TABLE 3:** The Influence of attachment avoidance on SNS Addiction with gender and anxiety as covariates.

	**Consequent**					
**Antecedent**	**Model 1 (ERD)**			**Model 2 (SNS addiction)**		
	**Coefficient**	**SE**	***p***	**Coefficient**	**SE**	***p***
Avoidance attachment	0.14	0.11	=0.2	−0.08	0.16	=0.63
ERD	–	–	–	0.60	0.07	<0.001
Gender	2.71	1.16	<0.05	3.94	1.77	<0.05
Anxiety attachment	0.96	0.11	<0.001	0.9	0.17	<0.001
Constant	14.46	3.05	<0.001	35.17	4.72	<0.001
	*R*^2^ = 0.18			*R*^2^ = 0.281		
	*F*(3,459) = 34.2,*p* < 0.001			*F*(4,458) = 44.7, *p* < 0.001		

*ERD, emotional regulation difficulty.*

## Discussion

The present study examined the mediating role of emotion dysregulation on the association between insecure attachment and SNS addiction. We found that attachment anxiety positively predicts SNS addiction, while attachment avoidance does not. Emotional dysregulation partly mediated the relationship between anxiety attachment and SNS addiction, but it did not mediate the relationship between avoidance attachment and SNS addiction. This study is, to the best of our knowledge, the first to investigate the correlation between attachment style, emotion dysregulation, and SNS addiction among college students.

Our results align with those of prior studies indicating that attachment anxiety is a predictor of SNS addiction (Wardecker et al., 2016; Blackwell et al., 2017; Lucia et al., 2017; Flynn et al., 2018; Chen, 2019). Individuals with high levels of attachment anxiety tend to have a stronger need for social belongingness, feedback, and comfort, all of which can be fulfilled to some extent by SNSs (Hart et al., 2015). Indeed, other studies revealed that SNS use is closely correlated with individual’s attachment characteristics (Kowert and Oldmeadow, 2015; Lin, 2015; Reed et al., 2015). An initial study found that individuals with high attachment anxiety were more likely to spend more time on an SNS site, use it when they were feeling negative emotions, and show concern over how other Facebook users perceived them (Oldmeadow et al., 2013).

Extant studies have shown that people high in emotion dysregulation often use the internet for mood alteration, which can in turn lead to internet addiction (Lei and Wu, 2007). The same may be true of SNSs. We found that attachment anxiety predicts emotional regulation difficulty, which in turn predicts SNS addiction. That is, emotional regulation difficulty mediates the relationship between attachment anxiety and SNS addiction. This accords with an attachment and emotion regulation perspective. Individuals with high attachment anxiety tended to up-regulate their emotions, thereby overreacting and maintaining high levels of negative emotion. Thus, they may resort to SNS to regulate their emotions through interaction on SNS. It is perhaps this feature that is particularly attractive to users high in attachment anxiety.

We did not find a positive relationship between avoidance attachment and SNS addiction. Results from prior studies on the predictive role of attachment avoidance for SNS addiction are mixed. While one study found that attachment avoidance positively predicted SNS addiction (Blackwell et al., 2017), another study found that it negatively predicted SNS addiction (Worsley et al., 2018b). However, our results agree with one study showing that avoidance attachment is associated with lower SNS use (Oldmeadow et al., 2013). Due to the difference in research focus, however, our study cannot be directly compared with those two previous studies. It is reasonable that attachment avoidance does not predict SNS addiction, given that individuals with an avoidant attachment style may find close relationships through SNS to be distressing, and they may find it difficult to engage in self-disclosure and displays of warmth toward others.

This study’s limitations include. First, the imbalance in gender should be avoided in future studies. Second, the cross-sectional nature of the investigation makes it impossible to judge the causality of the relationships. Third, the use of self-report measurements may threaten the validity of the relationship between variables. Last, the conclusions were draw from college student, whether it can be generalized to population groups need further investigation. Despite these limitations, our study adds to previous findings by showing that attachment theory is useful for explaining the nature of excessive SNS use, not just SNS use in general (Hart et al., 2015). Future research aiming to develop treatments or interventions for SNS addiction should consider focusing on emotion regulation and insecure attachment, as these both appear to be risk factors of SNS addiction.

## Data Availability Statement

The raw data supporting the conclusions of this manuscript will be made available by the authors, without undue reservation, to any qualified researcher.

## Ethics Statement

All the data were collected in August 2018. Approval for the study was obtained from the Human Research Ethics Committee of Chongqing University of Posts and Telecommunications. Written informed consent was obtained from all participants above the age of 16 and from the parents of participants below the age of 16. They were informed that their participation was voluntary and that they could terminate participation anytime they wanted. Participants received no rewards for their participation.

## Author Contributions

CL designed the study. Both authors collected the data. J-LM wrote the manuscript.

## Conflict of Interest

The authors declare that the research was conducted in the absence of any commercial or financial relationships that could be construed as a potential conflict of interest.

## Supplementary Material

TABLE S1 The data of the study are presented here. It includes gender, age, avoidance, anxiety, and social networking sites addiction score of each participant.
